# A Self-Care Mobile Application for Patients with Acute Decompensated Heart Failure During the COVID-19 Pandemic

**DOI:** 10.3390/jcm15208183

**Published:** 2026-10-21

**Authors:** Hani Essa, Gregory Yoke Hong Lip, Rajiv Sankaranarayanan

**Affiliations:** 1 Department of Cardiology, Aintree University Hospital, Liverpool University Hospitals NHS Foundation Trust, Lower Lane, Liverpool L9 7AL, UK; hani.essa@liverpool.ac.uk; 2 Institute of Life Course and Medical Sciences, University of Liverpool, Liverpool L7 8TX, UK; 3 Department of Clinical Medicine, Aalborg University, 9000 Aalborg, Denmark; 4 Department of Cardiology, Lipidology and Internal Medicine, Medical University of Bialystok, 15-540 Bialystok, Poland

**Keywords:** acute decompensated heart failure, mobile health (mHealth), self-care, ambulatory heart failure care

## Abstract

**Background:** Mobile health applications may support self-care following acute decompensated heart failure (ADHF), particularly when conventional healthcare contact is limited. We evaluated the integration of the Aintree Heart Failure Passport (AHFP) application into a specialist ambulatory ADHF pathway and described self-care behaviour, medication adherence and 30-day heart failure rehospitalisation among app users. **Methods:** This prospective, single-centre observational study included consecutive patients treated with day-case intravenous diuretics in a consultant-led ambulatory heart failure unit at a UK university hospital between March 2020 and July 2021. Patients with access to a smartphone were offered the AHFP application. Self-care behaviour and medication adherence were assessed two weeks after presentation using the nine-item European Heart Failure Self-care Behaviour Scale (EHFScB-9) and five-item Medication Adherence Report Scale (MARS-5), respectively. Thirty-day HF hospitalisation among application users was compared with a separate cohort of hospitalised HF patients recorded in the National Heart Failure Audit (73/382, 19.1%). This inpatient cohort was used as a contextual benchmark rather than a matched control group. **Results:** Of 221 consecutive patients treated for ADHF, 148 (67%) downloaded and registered with the AHFP application. Their mean age was 62 ± 6.1 years, and 45% were women. At two weeks, the mean EHFScB-9 score was 19.1 ± 6.7, and the mean MARS-5 score was 23.3 ± 1.0. Within 30 days, 16 patients (10.8%; 95% CI 6.8–16.8%) were hospitalised with HF, accounting for 73 inpatient bed-days. The corresponding 30-day readmission rate in the separate inpatient audit cohort was 19.1% (73/382). **Conclusions:** The AHFP application was downloaded and registered by 67% of patients treated within an ambulatory ADHF pathway. Self-care behaviour and medication adherence were assessed at two weeks. The observed 30-day HF hospitalisation rate was lower than that of a separate inpatient audit cohort, although differences in patient selection and care setting preclude attribution to the application. These findings support the feasibility of integrating a self-care application into specialist ambulatory HF care and warrant further evaluation in controlled studies.

## 1. Introduction

Heart failure (HF) remains a major cause of morbidity, mortality and healthcare utilisation worldwide. In the United Kingdom, approximately one million people live with HF, while recurrent hospitalisation following an episode of acute decompensated heart failure (ADHF) remains common [1,2]. Hospital admissions account for a substantial proportion of the economic burden associated with HF, making strategies that prevent avoidable deterioration and rehospitalisation an important clinical priority [1,2]. Congestion frequently develops progressively over several days, with increasing peripheral oedema, breathlessness and weight gain. This provides an opportunity for patients to recognise deterioration earlier and seek intervention before emergency hospitalisation becomes necessary.

Effective HF self-care requires patients to monitor their symptoms and weight, adhere to prescribed treatment and recognise when deterioration should prompt contact with healthcare professionals. However, education delivered during clinical encounters may be difficult to retain and apply consistently after patients return home. Maintaining access to clear information and practical self-management advice between clinical contacts may therefore support patients in responding appropriately to changes in their condition.

Mobile health applications offer a potentially scalable means of extending HF education and self-management support beyond conventional healthcare encounters. They can combine educational resources, symptom and weight monitoring, medication reminders and guidance about when to seek professional assistance. Their potential value became particularly apparent during the COVID-19 pandemic, when face-to-face healthcare interactions were disrupted and greater reliance was placed on remote and ambulatory models of care [3]. However, the uptake and potential value of such applications when incorporated into established ambulatory ADHF pathways require further evaluation.

The Aintree Heart Failure Passport (AHFP) is a freely available smartphone application developed to reinforce HF education, self-care and medication adherence. It provides traffic-light-based symptom guidance, weight monitoring, medication and appointment reminders, educational videos and a mood-screening function. The application was designed to complement multidisciplinary HF care by providing patients with continued access to education and self-management support between clinical encounters.

We therefore prospectively evaluated uptake of the AHFP, defined as application download and registration, among consecutive patients treated for ADHF within a specialist ambulatory HF service. We also assessed self-reported self-care behaviour and medication adherence two weeks after presentation and examined 30-day HF hospitalisation among patients who registered with the application. The observed hospitalisation rate was contextualised against the 30-day readmission rate of a separate inpatient HF cohort recorded in the National Heart Failure Audit.

## 2. Methods

### 2.1. Study Design and Setting

This prospective, single-centre observational service evaluation was conducted at a UK university hospital between March 2020 and July 2021, during the COVID-19 pandemic. Consecutive patients treated for acute decompensated heart failure (ADHF) within the Ambulatory Acute Heart Failure Unit (AAHFU) were assessed for eligibility for the Aintree Heart Failure Passport (AHFP) application.

The AAHFU is a consultant-led multidisciplinary day-case service delivered predominantly by HF specialist nurses. It provides specialist assessment and treatment for selected patients with worsening congestion, including bolus intravenous loop diuretic therapy. This model enables patients to receive treatment for ADHF without conventional inpatient admission where clinically appropriate.

The study evaluated uptake of the AHFP, self-reported HF self-care behaviour and medication adherence at two weeks, and HF-related rehospitalisation within 30 days of the index presentation.

### 2.2. AHFP Intervention

The AHFP is available free of charge on iOS and Android platforms and was used in addition to the established ambulatory HF pathway. Its self-management functions include

A traffic-light-based symptom tracker providing corresponding self-care and escalation advice.

Daily weight monitoring, with prompts to contact the HF team when weight gain suggests worsening fluid retention.

Timed medication and healthcare-appointment reminders.

Educational videos addressing HF and its management.

A mood-check questionnaire screening for symptoms of anxiety or depression.

The application was developed with input from a multidisciplinary team comprising HF consultant cardiologists, specialist nurses, cardiology fellows, a cardiac pharmacist, a consultant pharmacologist and an expert patient. Application uptake was defined as downloading and registering with the AHFP application. Objective measures of subsequent application engagement, including frequency of use, duration of use and utilisation of individual functions, were not collected.

### 2.3. Participants

- Patients were eligible if ADHF was the primary diagnosis, as determined by the treating HF team in accordance with the 2016 European Society of Cardiology guidelines for the diagnosis and treatment of acute and chronic heart failure [4]. Participants were required to be able to speak, read and understand English and to have access to a compatible smartphone. Patients without their own smartphone were eligible if a carer had access to a suitable device and was willing to assist with application use.
- Patients with cognitive impairment and those receiving palliative care were excluded. Patients without personal or carer access to a smartphone were ineligible for the digital intervention.

### 2.4. Outcomes and Data Collection

- HF self-care behaviour and medication adherence were assessed two weeks after the index presentation by telephone or during clinical follow-up.
- Self-care behaviour was evaluated using the validated nine-item European Heart Failure Self-care Behaviour Scale (EHFScB-9) [5]. The total score ranges from 9 to 45, with lower scores indicating better self-care behaviour. Medication adherence was assessed using the five-item Medication Adherence Report Scale (MARS-5) [6]. The total score ranges from 5 to 25, with higher scores indicating better reported adherence.

For contextual comparison, the observed hospitalisation rate was compared with an institutional cohort of 382 patients hospitalised with HF and recorded as part of the National Heart Failure Audit (NICOR), of whom 73 (19.1%) were readmitted within 30 days of discharge. This represented a separate inpatient population rather than the 73 patients treated within the ambulatory pathway who did not use the application. As the comparator consisted of hospitalised patients rather than an equivalent ambulatory population, it was considered an institutional benchmark rather than a matched control group.

### 2.5. Statistical Analysis

Continuous variables were summarised as means with standard deviations, while categorical variables were reported as frequencies and percentages. The 30-day HF hospitalisation rate among AHFP users was presented with its 95% confidence interval. The observed proportions in the AHFP cohort (16/148) and the institutional inpatient audit cohort (73/382) were compared using an uncorrected Pearson chi-squared test based on a 2 × 2 contingency table. All tests were two-sided, with *p* < 0.05 considered statistically significant. The comparison was exploratory and unadjusted, given the differences in patient selection and care setting between the two cohorts.

### 2.6. Ethical Considerations

The AHFP application was funded by the hospital. This project was registered as a service evaluation through the hospital’s institutional governance process. The evaluation was conducted in accordance with applicable institutional governance requirements.

## 3. Results

### 3.1. Study Population

**Figure 1:**
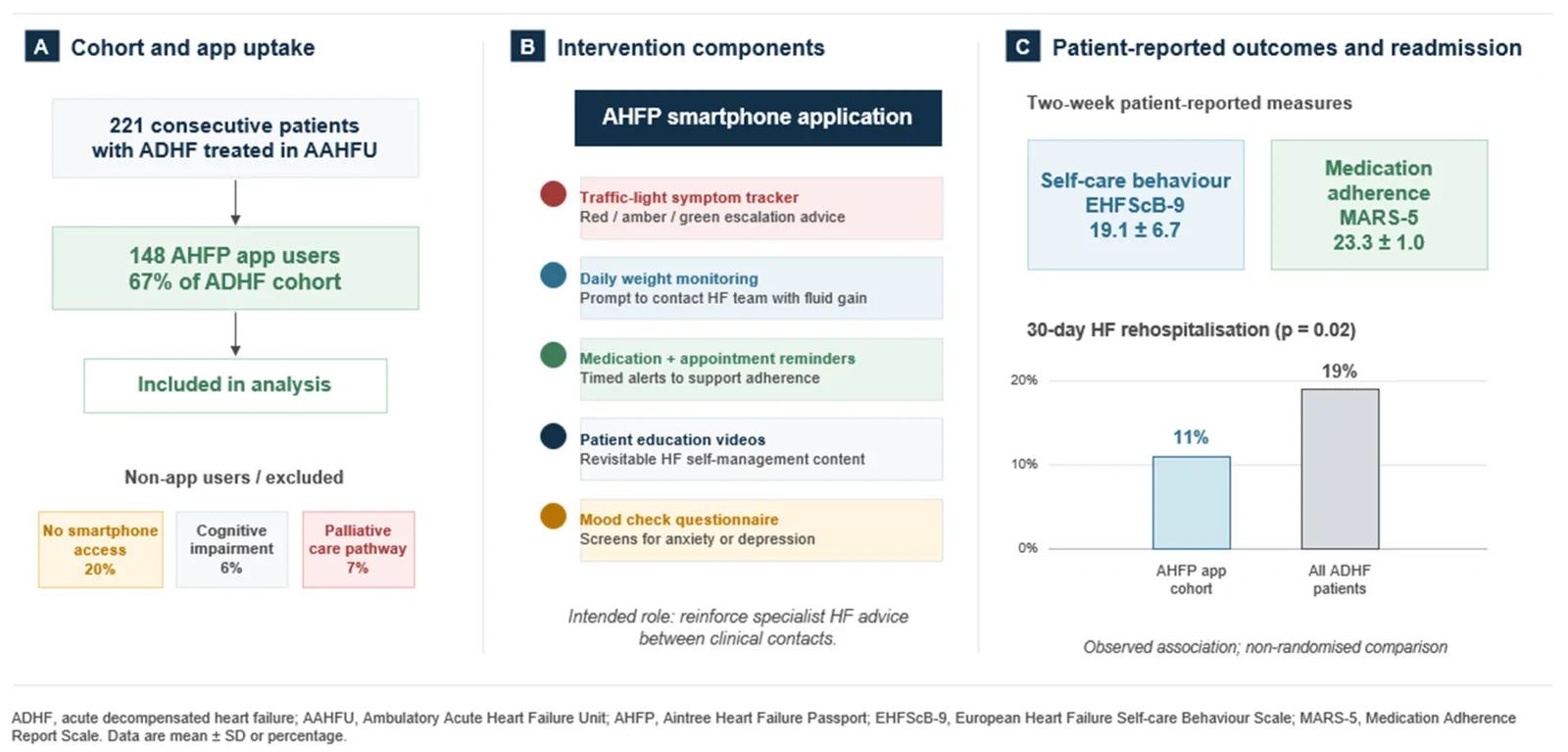
Aintree Heart Failure Passport (AHFP) study overview and outcomes. Of 221 patients with acute decompensated heart failure, 148 (67%) used the AHFP app. App users demonstrated high self-care (EHFScB-9 19.1 ± 6.7) and medication adherence (MARS-5 23.3 ± 1.0). Thirty-day HF rehospitalisation was 11% versus 19% in the wider institutional ADHF population (*p* = 0.02).

During the study period, 221 consecutive patients were treated for ADHF in the AAHFU (Figure 1). Of these, 148 (67%) downloaded and registered with the AHFP application and were included in the analysis. The remaining 73 patients (33%) did not enter the application cohort. Reasons for non-inclusion included lack of smartphone access (*n* = 44, 20%), cognitive impairment (*n* = 13, 6%), and receipt of palliative care (*n* = 16, 7%), expressed as proportions of the overall cohort.

The mean age of patients included in the application cohort was 62 ± 6.1 years, and 45% were women. Heart failure with reduced ejection fraction (HFrEF) was present in 55% of patients, while 34% had heart failure with preserved ejection fraction (HFpEF) and 11% had heart failure with mildly reduced ejection fraction (HFmrEF).

Despite the relatively young mean age of the cohort, participants had a substantial burden of comorbidity. The mean Charlson Comorbidity Index was 4.9 ± 1.0, indicating a moderate-to-high comorbidity burden. The mean Rockwood Clinical Frailty Scale score was 4.7 ± 0.7, suggesting that the cohort was, on average, between vulnerable and mildly frail.

### 3.2. Self-Care Behaviour and Medication Adherence

At the two-week follow-up assessment, the mean European Heart Failure Self-care Behaviour Scale (EHFScB-9) score was 19.1 ± 6.7, with lower scores indicating better self-care behaviour. The mean Medication Adherence Report Scale (MARS-5) score was 23.3 ± 1.0, with higher scores indicating better reported adherence. The MARS-5 score was close to the maximum possible score of 25. Both measures were self-reported and assessed at a single time point, without baseline measurements or a control group.

Reported medication adherence was similarly high, with a mean five-item Medication Adherence Report Scale (MARS-5) score of 23.3 ± 1.0. Collectively, these findings indicated favourable self-reported self-care behaviour and medication adherence among patients using the AHFP application following treatment for ADHF.

### 3.3. Thirty-Day Heart Failure Rehospitalisation

Within 30 days following ambulatory treatment, 16 of the 148 patients who downloaded and registered with the AHFP application were hospitalised because of HF, corresponding to a 30-day HF hospitalisation rate of 10.8% (95% CI 6.8–16.8%). These hospitalisations accounted for a total of 73 inpatient bed-days.

For contextual comparison, the 30-day readmission rate among hospitalised HF patients recorded in the institutional National Heart Failure Audit cohort was 19.1% (73/382). The absolute difference between the two observed proportions was 8.3 percentage points, with an unadjusted Pearson chi-squared test yielding χ^2^ = 5.258 (*p* = 0.022). However, the two cohorts represented different clinical populations, with patients in the audit cohort receiving inpatient rather than ambulatory treatment. The comparison was therefore exploratory and does not establish an association attributable to application use.

## 4. Discussion

In this prospective single-centre study, approximately two-thirds of consecutive patients treated within an ambulatory ADHF pathway downloaded and registered with the AHFP application. At two-week follow-up, patients reported self-care behaviour and medication adherence scores of 19.1 ± 6.7 and 23.3 ± 1.0, respectively. The observed 30-day HF hospitalisation rate was 10.8%, compared with a 19.1% readmission rate among 382 hospitalised HF patients recorded in the institutional National Heart Failure Audit. However, these cohorts differed in their clinical setting and patient selection, limiting the interpretation of this comparison. The findings demonstrate that a dedicated self-care application can be incorporated into an established ambulatory HF service, although its independent clinical benefit remains uncertain.

Self-care and medication adherence are important components of HF management, as patients must recognise worsening symptoms, monitor their weight, adhere to prescribed treatment and know when to contact their HF team. The AHFP was designed to reinforce these behaviours through symptom and weight guidance, medication reminders and educational resources. The mean EHFScB-9 score was 19.1 ± 6.7, with lower scores indicating better self-care behaviour, while the mean MARS-5 score was 23.3 ± 1.0, close to the maximum possible score of 25. The latter raises the possibility of a ceiling effect, limiting the ability of the scale to distinguish between patients reporting relatively high adherence. Both measures relied on self-report and may therefore have been affected by recall and social desirability bias. Furthermore, neither measure was assessed before application registration or in a contemporaneous control group. It is therefore not possible to determine whether these scores reflected an improvement following use of the application or pre-existing characteristics of patients who were willing and able to engage with digital technology.

The observed 30-day HF hospitalisation rate of 10.8% among AHFP participants was lower than the 19.1% readmission rate recorded among hospitalised HF patients in the institutional National Heart Failure Audit. Although this difference was statistically significant in an unadjusted comparison (*p* = 0.022), the two populations were not directly comparable. Patients selected for ambulatory treatment may differ substantially from those requiring inpatient admission in terms of acute illness severity, frailty, comorbidity and baseline prognosis. Furthermore, patients with access to a smartphone who registered with the application may have differed in health literacy, social support and capacity for self-management. The audit population was not prospectively matched to the AHFP cohort, and no adjustment was made for these differences. The observed difference therefore cannot be attributed to the application and should be regarded as hypothesis-generating rather than evidence of clinical effectiveness.

The application was also delivered within a consultant-led, specialist nurse-delivered ambulatory service. The potential contribution of the AHFP cannot be separated from the effects of close clinical follow-up, multidisciplinary decision-making and rapid access to intravenous diuretic treatment. The findings should consequently be interpreted as reflecting an app-supported ambulatory pathway rather than the independent effect of the application. The rehospitalisation comparison is best regarded as hypothesis-generating and requires confirmation in a controlled study.

The study also identified practical limitations to the implementation of digital HF care. One fifth of the consecutive ADHF population lacked access to a smartphone, even when access through a carer was permitted. Cognitive impairment prevented participation among a further proportion of patients. These barriers are particularly relevant in HF, which commonly affects older adults with multimorbidity, frailty, sensory impairment or limited digital literacy. Digital interventions could widen existing inequalities if they replace conventional care rather than supplement it. Future implementation should therefore preserve non-digital routes to education and clinical support while considering carer involvement, simplified interfaces, accessibility requirements and availability in languages other than English.

Digital interventions offer several potential advantages in HF management, although their effectiveness appears to depend on the nature and intensity of the intervention. Nurse-led self-care education has previously been associated with improved clinical outcomes, while an integrative review of mobile applications identified encouraging evidence regarding HF self-management [7,8]. More recently, the SMART-HF randomised trial evaluated a smartphone application combined with Bluetooth-connected monitoring devices and a feedback system [9]. Although improvements in dyspnoea scores were reported, there was no significant difference in the composite outcome of death, rehospitalisation or urgent HF visits. Similarly, TIM-HF2 evaluated a structured telemedical management programme involving remote monitoring and clinical intervention, representing a substantially different approach from the educational and self-management functions of the AHFP [10]. During the COVID-19 pandemic, Mariani et al. also demonstrated the feasibility and acceptability of virtual consultations in patients receiving electrophysiology care, highlighting the wider potential for remote cardiovascular healthcare [11]. However, these interventions differ considerably in their components, intensity and target populations. Their findings therefore cannot be directly extrapolated to the AHFP, which was designed to complement existing specialist ambulatory care rather than provide independent remote clinical monitoring.

The COVID-19 pandemic provided an important context for this study. Restrictions on conventional healthcare interactions increased the need for ambulatory treatment and remote self-management support. The combination of day-case treatment for worsening congestion and continued access to digital education offered a pragmatic means of maintaining HF care while reducing reliance on inpatient admission. Conversely, pandemic-related changes in healthcare-seeking behaviour, admission thresholds and the availability of community services may have influenced the observed rehospitalisation rate. Further evaluation outside the pandemic setting is necessary to determine whether similar uptake and outcomes can be achieved during routine service delivery.

This study has several strengths. It prospectively evaluated a freely available application within a real-world ambulatory ADHF service, considered consecutive patients for participation and used validated measures of self-care behaviour and medication adherence. Recording reasons for non-participation also provided useful information about barriers to digital implementation. However, several limitations should be acknowledged. The relatively small, single-centre design limits generalisability, while the absence of randomisation or a matched control group prevents causal conclusions regarding clinical outcomes. In particular, the use of a separate hospitalised HF population as a comparator introduces substantial potential for selection bias and confounding. Differences in baseline clinical characteristics, treatment setting and potentially outcome ascertainment may have influenced the observed hospitalisation rates. Furthermore, application uptake was defined by download and registration, and no objective measures of subsequent engagement were available. Self-care behaviour and medication adherence were assessed at a single time point using self-reported questionnaires, without baseline measurements or a control group. The short follow-up period, restriction to English-speaking patients and exclusion of patients with cognitive impairment further limit the applicability of these findings to the wider HF population.

Future studies should include baseline and longitudinal assessments, objective measures of application engagement and an appropriately matched or randomised control group. Outcomes should extend beyond short-term rehospitalisation to include mortality, quality of life, medication adherence, healthcare utilisation and cost-effectiveness. Evaluations should also examine which application functions are most useful and whether accessible design, carer-supported use and alternative methods of delivery can reduce digital exclusion.

## 5. Conclusions

A dedicated HF self-care application was successfully integrated into a specialist ambulatory ADHF pathway and was associated with favourable self-reported self-care behaviour and medication adherence. The lower 30-day HF rehospitalisation rate observed among app users is encouraging but remains hypothesis-generating because of the non-randomised design and potential selection bias. Larger controlled studies are required to determine the clinical effectiveness, cost-effectiveness and accessibility of app-supported HF care.

## Data Availability

Data available on reasonable request.

## References

[1] Shahim B., Kapelios C.J., Savarese G., Lund L.H. (2023). Global Public Health Burden of Heart Failure: An Updated Review. Card. Fail. Rev..

[2] Cowie M.R., Anker S.D., Cleland J.G.F., Felker G.M., Filippatos G., Jaarsma T., Jourdain P., Knight E., Massie B., Ponikowski P. (2014). Improving care for patients with acute heart failure: Before, during and after hospitalization. ESC Heart Fail..

[3] Mafi J.N., Craff M., Vangala S., Pu T., Skinner D., Tabatabai-Yazdi C., Nelson A., Reid R., Agniel D., Tseng C.H. (2022). Trends in US Ambulatory Care Patterns During the COVID-19 Pandemic, 2019–2021. JAMA.

[4] Ponikowski P., Voors A.A., Anker S.D., Bueno H., Cleland J.G., Coats A.J., Falk V., González-Juanatey J.R., Harjola V.P., Jankowska E.A. (2016). 2016 ESC Guidelines for the diagnosis and treatment of acute and chronic heart failure: The Task Force for the diagnosis and treatment of acute and chronic heart failure of the European Society of Cardiology (ESC). Developed with the special contribution of the Heart Failure Association (HFA) of the ESC. Eur. J. Heart Fail..

[5] Jaarsma T., Arestedt K.F., Mårtensson J., Dracup K., Strömberg A. (2009). The European Heart Failure Self-care Behaviour scale revised into a nine-item scale (EHFScB-9): A reliable and valid international instrument. Eur. J. Heart Fail..

[6] Chan A.H.Y., Horne R., Hankins M., Chisari C. (2020). The Medication Adherence Report Scale: A measurement tool for eliciting patients’ reports of nonadherence. Br. J. Clin. Pharmacol..

[7] Son Y.J., Choi J., Lee H.J. (2020). Effectiveness of Nurse-Led Heart Failure Self-Care Education on Health Outcomes of Heart Failure Patients: A Systematic Review and Meta-Analysis. Int. J. Environ. Res. Public Health.

[8] Athilingam P., Jenkins B. (2018). Mobile Phone Apps to Support Heart Failure Self-Care Management: Integrative Review. JMIR Cardio.

[9] Yoon M., Lee S., Choi J.Y., Jung M.H., Youn J.C., Shim C.Y., Choi J.O., Kim E.J., Kim H., Yoo B.S. (2024). Effectiveness of a Smartphone App-Based Intervention With Bluetooth-Connected Monitoring Devices and a Feedback System in Heart Failure (SMART-HF Trial): Randomized Controlled Trial. J. Med. Internet Res..

[10] Koehler F., Koehler K., Deckwart O., Prescher S., Wegscheider K., Kirwan B.A., Winkler S., Vettorazzi E., Bruch L., Oeff M. (2018). Efficacy of telemedical interventional management in patients with heart failure (TIM-HF2): A randomised, controlled, parallel-group, unmasked trial. Lancet.

[11] Mariani M.V., Pierucci N., Forleo G.B., Schiavone M., Bernardini A., Gasperetti A., Mitacchione G., Mei M., Giunta G., Piro A. (2023). The Feasibility, Effectiveness and Acceptance of Virtual Visits as Compared to In-Person Visits among Clinical Electrophysiology Patients during the COVID-19 Pandemic. J. Clin. Med..

